# Towards attainment of the 2030 goal for childhood cancer survival for the World Health Organization Global Initiative for Childhood Cancer: An ecological, cross-sectional study

**DOI:** 10.1371/journal.pgph.0002530

**Published:** 2024-08-19

**Authors:** Emily R. Smith, Cesia Cotache-Condor, Harold Leraas, Paul Truche, Zachary J. Ward, Cristina Stefan, Lisa Force, Nickhill Bhakta, Henry E. Rice

**Affiliations:** 1 Duke Global Health Institute, Durham, North Carolina, United States of America; 2 Duke Center for Global Surgery and Health Equity, Duke University, Durham, North Carolina, United States of America; 3 Department of Emergency Medicine, Duke University, Durham, North Carolina, United States of America; 4 Department of Surgery, Duke School of Medicine, Duke University, Durham, North Carolina, United States of America; 5 Department of Surgery, Rutgers University, New Brunswick, New Jersey, United States of America; 6 Center for Health Decision Science, Harvard T.H. Chan School of Public Health, Boston, Massachusetts, United States of America; 7 Singhealth Duke-National University of Singapore, Singapore, Singapore; 8 Department of Health Metrics Sciences and Department of Pediatrics, Division of Pediatric Hematology/Oncology, University of Washington, Seattle, Washington, United States of America; 9 Department of Global Pediatric Medicine, St. Jude Children’s Research Hospital, Memphis, Tennessee, United States of America; African Population and Health Research Center, KENYA

## Abstract

The World Health Organization (WHO) recently launched the Global Initiative for Childhood Cancer (GICC), with the goal of attaining at least 60% cancer survival for children worldwide by the year 2030. This study aims to describe the global patterns of childhood cancer survival in 2019 to help guide progress in attaining the GICC target goal. In this ecological, cross-sectional study, we used 5-year net childhood cancer survival (2015–2019) data from a prior micro-modeling study from 197 countries and territories. Descriptive statistics were used to analyze the patterns of overall childhood cancer survival and survival for each of the six cancer tracer diagnoses as proposed by the GICC. We used hot spot analysis to identify geographic clusters of high and low cancer survival. Most high-income countries reached at least 60% (92%, n = 59/64), net childhood cancer survival at baseline. No lower-middle-income or low-income country reached at least 60% overall cancer survival at baseline. The South-East Asia region had the highest proportion of countries that did not achieve at least 60% survival at baseline (100%, n = 10/10), followed by the African region (98%, n = 49/50). For each cancer tracer diagnosis, we found the highest number of countries that have achieved at least 60% survival was for Burkitt lymphoma (44%, n = 87/197) followed by acute lymphocytic leukemia (41%, n = 80/197).Hot spot analysis showed the highest overall survival was concentrated in North America and Europe, while the lowest survival was concentrated in Sub-Saharan Africa and South-East Asia.A majority of LMICs had not reached the WHO target goal of at least 60% survival from childhood cancer at baseline in 2019, with variable success for the six childhood cancer tracer diagnoses of the GICC. These findings provide baseline assessment of individual country performance to help achieve the GICC goal of 60% overall cancer survival globally by 2030.

## Introduction

The Lancet Oncology Commission on Sustainable Care for Children with Cancer has estimated that between 2020 and 2050, over 11 million children will die from cancer worldwide if no additional investments are made to improve cancer care systems [1]. The cancer burden in children is disproportionally concentrated in low- and middle-income countries (LMICs), where 85–90% of cancer of total cancer cases [2, 3]. There are wide disparities in survival in children with cancer globally, with 20% survival for children with cancer in LMICs compared to 80–85% survival in high-income countries (HICs) [4, 5]. In light of these disparities, in 2018 the World Health Organization (WHO) launched the Global Initiative for Childhood Cancer (GICC), with the goal of attaining at least 60% cancer survival for children worldwide by the year 2030 [6].

According to GICC and its CureAll operational framework, six common cancer diagnoses, including acute lymphoblastic leukemia (ALL), Burkitt lymphoma, Hodgkin’s lymphoma, retinoblastoma, Wilms tumor, and low-grade central nervous system glioma, were chosen as tracer diagnoses to monitor overall progress towards the GICC goal [7]. Each cancer tracer is highly curable with proven therapies and has a unique feature for which it was selected [7–10]. For example, ALL is the most prevalent childhood cancer globally, Burkitt lymphoma is prevalent in many LMICs, Hodgkin lymphoma is common in adolescents, retinoblastoma is highly curable with early diagnosis, and Wilms tumor and low-grade glioma require robust systems of multidisciplinary care.

Five years after the launch of the GICC, only a few countries have reported baseline observed data of survival for all or some of the six cancer tracers [11–13]. Some of these reports are limited by the variability of the metrics used to report survival. For instance, Morocco reported a 3-year survival analysis at baseline, while Malawi and Uganda reported 2-year and 3-year survival, respectively [12, 13]. Moreover, there is not yet a global baseline report upon which monitoring, and evaluation of the initial goals established by the GICC can be made. Therefore, this study aims to describe the global patterns of overall childhood cancer survival and the six childhood cancer tracer diagnoses proposed by GICC at the beginning of the launch using descriptive and geospatial analysis. In doing so, our analyses will help define baseline status of countries and regions at the onset of the GICC initiative to help achieve 60% survival globally by 2030.

The Lancet Oncology Commission on Sustainable Care for Children with Cancer has estimated that between 2020 and 2050, over 11 million children will die from cancer worldwide if no additional investments are made to improve cancer care systems [1]. The incidence of childhood cancer is disproportionally concentrated in low- and middle-income countries (LMICs), which account for 85–90% of total cancer cases [2, 3]. There are wide disparities in survival in children with cancer globally, with 20% survival for children with cancer in LMICs compared to 80–85% survival in high-income countries [4, 5]. In light of these disparities, in 2018 the World Health Organization (WHO) launched the Global Initiative for Childhood Cancer (GICC), with the goal of attaining at least 60% cancer survival for children worldwide by the year 2030 [6].

According to GICC and its CureAll operational framework, six common cancer diagnoses, including acute lymphoblastic leukemia (ALL), Burkitt lymphoma, Hodgkin’s lymphoma, retinoblastoma, Wilms tumor, and low-grade central nervous system glioma (LGG), were chosen as tracer diagnoses to monitor overall progress towards the GICC goal [7]. Each cancer tracer is highly curable with proven therapies and has a unique feature for which it was selected [7–10]. For example, ALL is the most prevalent childhood cancer globally, Burkitt lymphoma is prevalent in many LMICs, Hodgkin lymphoma is common in adolescents, retinoblastoma is highly curable with early diagnosis, and Wilms tumor and LGG require robust systems of multidisciplinary care.

Five years after the launch of the GICC, only a few countries have reported baseline observed data of survival for all or some of the six cancer tracers [11–13]. Some of these reports are limited by the variability of the metrics used to report survival. For instance, Morocco reported a 3-year survival analysis at baseline, while Malawi and Uganda reported 2-year and 3-year survival, respectively [12, 13]. Moreover, there is not yet a global baseline report upon which monitoring, and evaluation of the initial goals established by the GICC can be made. Therefore, this study aims to describe the global patterns of overall childhood cancer survival and the six childhood cancer tracer diagnoses proposed by GICC at the beginning of the launch using descriptive and geospatial analysis. In doing so, our analyses will help define baseline status of countries and regions at the onset of the GICC initiative to help achieve 60% survival globally by 2030.

## Methods

### Study design and data collection

For this ecological, cross-sectional study, we collected mean estimates of overall childhood cancer net survival for 197 countries and territories from a previous modeling study for the 5-year period of 2015–2019 [14]. Three countries or territories (Cayman Islands, Faroe Islands, and Liechtenstein) were excluded since they did not have cancer survival information during that time. Childhood cancer was defined as all-inclusive cancers among children age 0–14 years old. Childhood cancer net survival estimates were also abstracted for each of the six cancer tracers diagnoses as suggested by the GICC, including ALL, Burkitt lymphoma, Hodgkin’s lymphoma, retinoblastoma, and Wilms tumor. Lymphoid cancers were used as a proxy for ALL, and the group of “other gliomas” was used as a proxy for LLG. We collected country income classification data for the year 2019 from the World Bank and WHO region classification from the WHO official website [15, 16].

### Data analysis

The 5-year net survival for childhood cancers overall and survival of each cancer tracer diagnosis were analyzed from two approaches. First, the original survival estimates were transformed into a dichotomous variable (60% or greater net survival, and less than 60% net survival) to reflect countries’ performance compared to the 2030 GICC global survival goal of 60%. Descriptive statistics were used to analyze a country’s survival for each cancer tracer. We used the chi-square test to compare survival across World Bank income level (low-income, low-middle income, upper-middle income, and high-income) and WHO region.

In a second approach, we used geospatial hot-spot analysis to identify geographic clusters of high and low 5-year cancer net survival overall as well as net survival for each cancer tracer diagnosis at a national level. This method identifies hot spots, defined as clusters of data points with adjacent data points with the highest childhood net survival, and cold spots, defined as clusters with adjacent data points with the lowest childhood net survival, by looking at each feature within the context of neighboring features. The Getis-Ord Gi* statistic was calculated for each feature in a dataset, with resultant z-scores and p-values indicating where features with either high or low values cluster spatially, and confidence levels of 90%, 95%, and 99%. The distance band to define neighboring features was set as the contiguity of edges and corners among countries. We generated geospatial analysis using ArcMap 10.3 (ESRI, Redlands, CA, USA).

## Results

**Fig 1:**
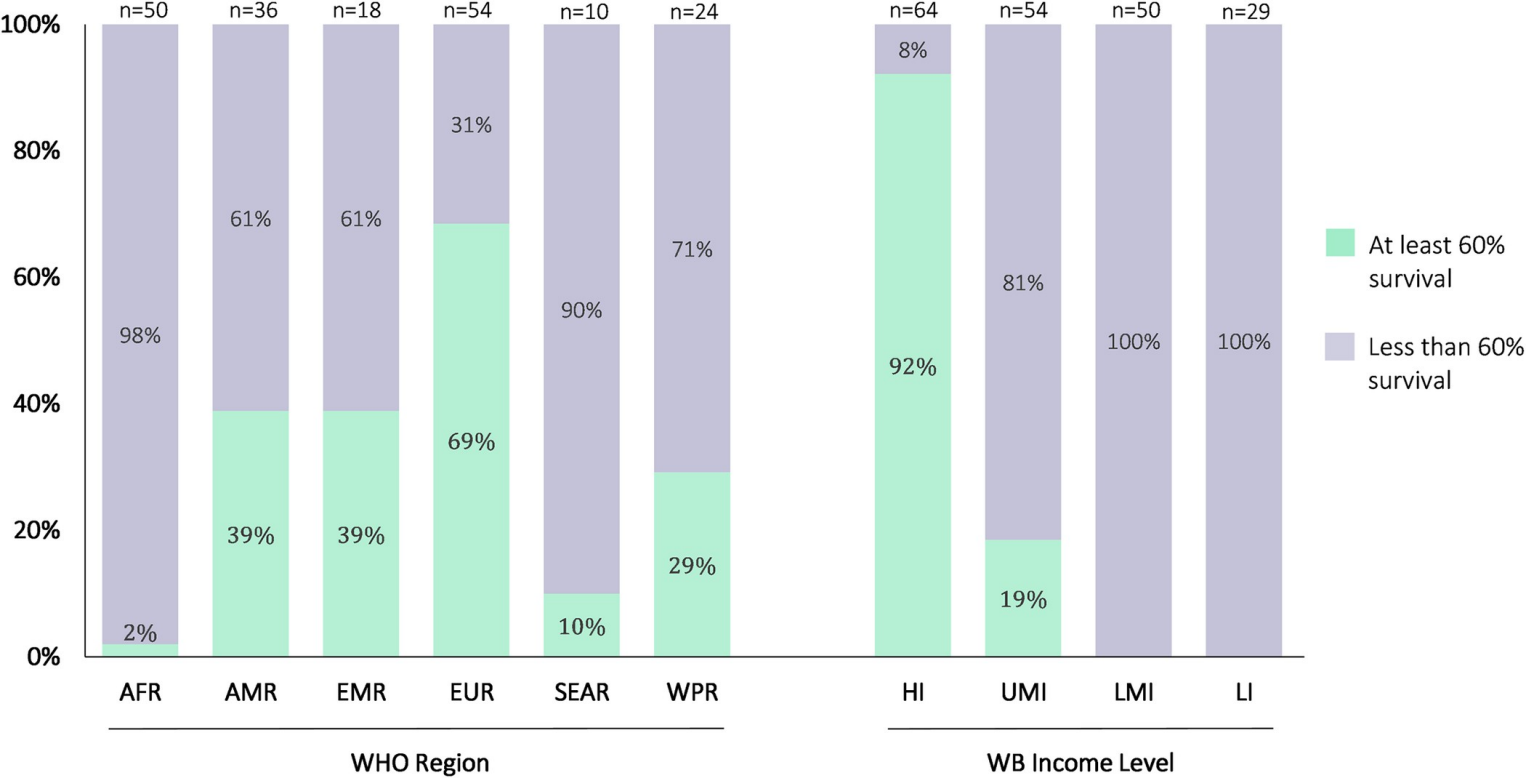
Status of childhood cancer 5-year (2015–2019) net survival overall at baseline and stratified by WHO region and World Bank income level. Note: 192 countries were included in the comparison by WHO region, as the other 5 areas were territories. The full 197 countries and territories were included when comparing by World Bank income levels. WPR = West Pacific Region, SEAR = South-East Asian Region, AMR = Region of the Americas, EUR = European Region, EMR = Eastern Mediterranean Region, AFR = African Region, HI = High income, UMI = Upper-middle income, LMI = Lower-middle income, LI = Low income.

A total of 197 countries and territories were included in this study. At baseline, 35% of countries (n = 69/197) had already reached the GICC goal of at least 60% net survival overall for childhood cancer in 2019 (**Fig 1**). The proportion of countries that reached at least 60% net survival overall differed by World Bank income classification (p <0.001) and WHO region (p <0.001). Most countries reaching at least 60% net survival overall were high-income countries (92%, n = 59/64), while the remaining were upper-middle-income countries (19%, n = 10/54). No low-income or lower-middle-income countries reached at least 60% net survival overall as of 2019. When stratified by WHO region, more than half of countries that reached at least 60% net survival overall were in the European Region (69%, n = 37/54), while only 2% (n = 1/50, Seychelles) of countries in the African region achieved at least 60% net survival overall. Between 10% and 39% of countries in the regions of South-East Asia, the Americas, the Easter-Mediterranean region, and the West Pacific regions achieved at least 60% net survival overall. The complete list of countries with status for net survival for childhood cancer overall and survival by each cancer tracer at baseline can be found in **S1 Table**.

**Fig 2:**
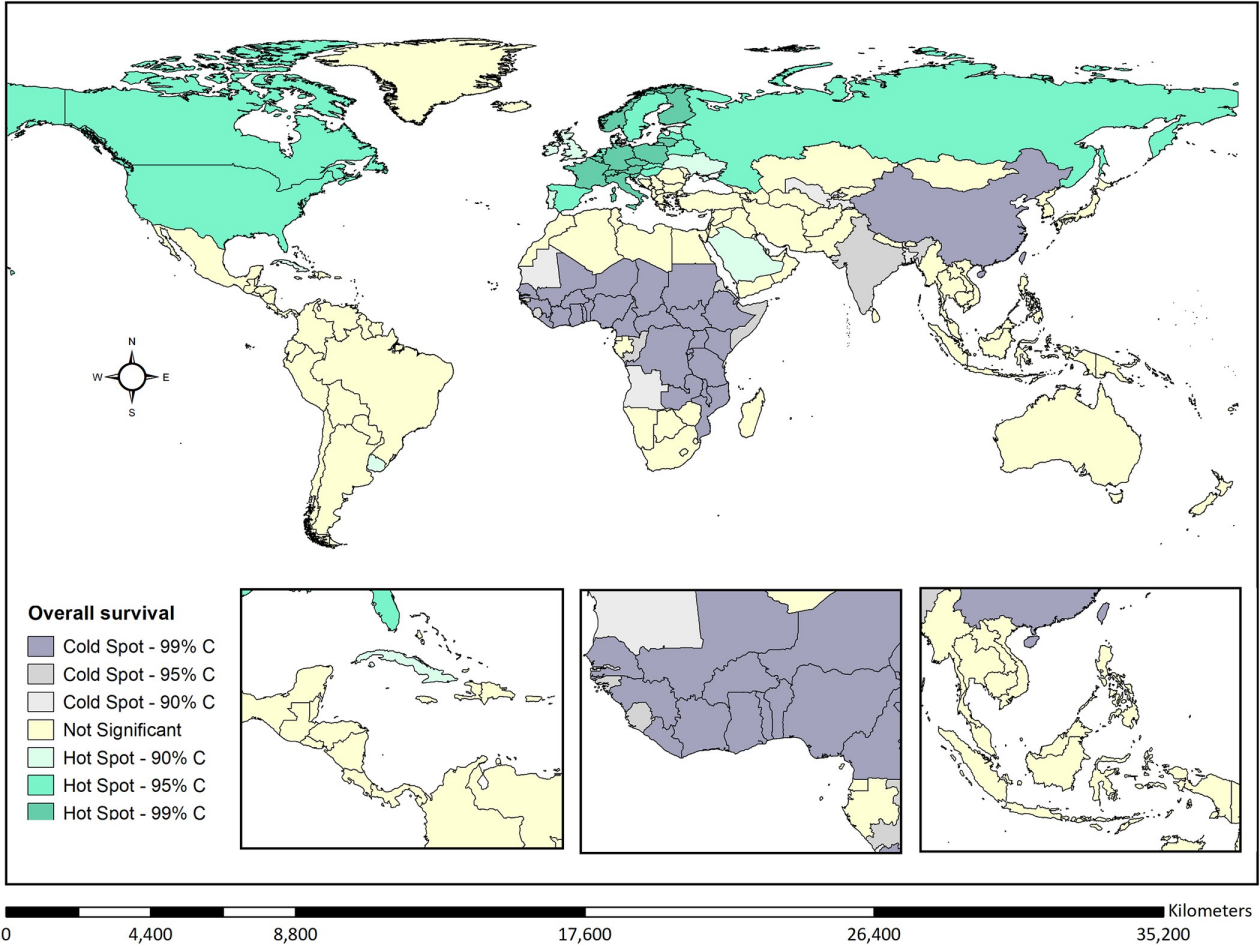
Hot spot analysis evaluating the geographic clusters with the highest and lowest childhood cancer 5-year (2015–2019) net survival overall. Hot spots (red) show clusters of data points with adjacent data points with high childhood survival. Cold spots (blue) show clusters with adjacent data points with low childhood survival. Base layer data available from the Humanitarian Data Exchange website: https://data.humdata.org/dataset/first-level-administrative-divisions-admin1.

Using hot spot analysis, we identified clusters of the highest net survival overall concentrated in the global north, including North America and Europe (**Fig 2**). Clusters with the lowest net survival overall were concentrated in the global south, including Sub-Saharan Africa and South-East Asia.

**Fig 3:**
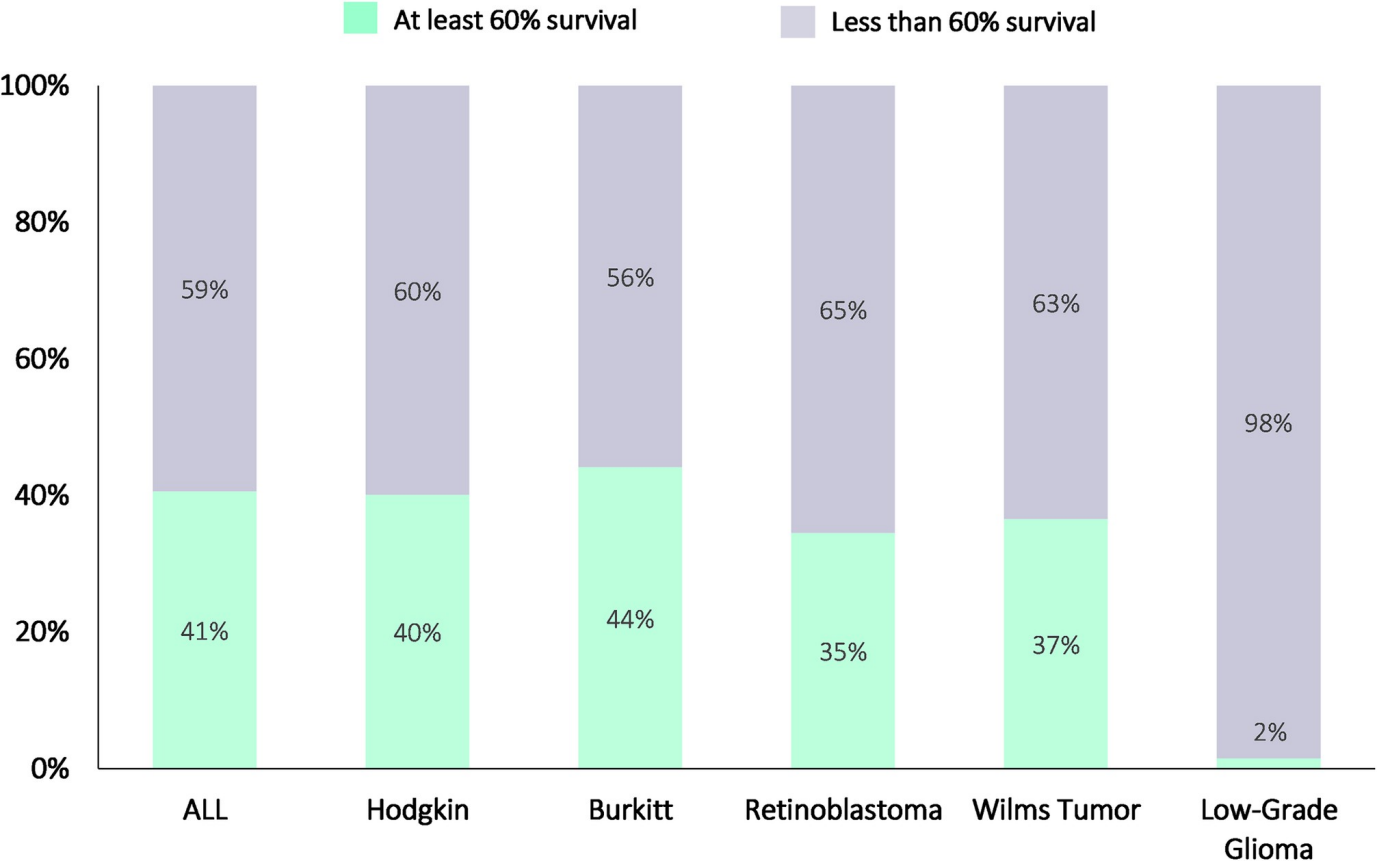
Status of childhood cancer 5-year (2015–2019) net survival at baseline by each of the six cancer tracer diagnoses. Note: ALL = Acute Lymphoblastic Leukemia. Lymphoid was used as a proxy of ALL. Other gliomas were used as a proxy for Low-Grade Glioma.

The analysis of survival for each cancer tracer found that the highest number of countries that achieved at least 60% net survival was found for Burkitt lymphoma (44%, n = 87/197), followed by ALL (41%, n = 80/197), Hodgkin’s lymphoma (40%, n = 79/197), Wilms tumor (37%, n = 72/197), and retinoblastoma (35%, n = 68/197). Only 2% (n = 3/197) of all countries achieved at least 60% net survival for LGG, using the proxy of ‘other gliomas.’ (**Fig 3**).

**Fig 4:**
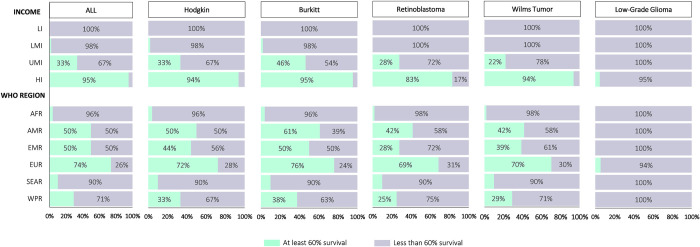
Status of childhood cancer 5-year (2015–2019) net survival at baseline by each of the six cancer tracer diagnoses and stratified made by WHO region and World Bank income level. Note: ALL = Acute Lymphoblastic Leukemia. Lymphoid was used as a proxy of ALL. Other gliomas were used as a proxy for Low-Grade Glioma.

When stratified by income level, the number of countries achieving at least 60% net survival for each cancer tracer, except for LGG, increased as income level increased (p <0.001). No low-income country achieved 60% net survival for all childhood cancer tracers (**Fig 4**). Only 2% (n = 1/50) of lower-middle-income countries achieved at least 60% net survival for ALL, Hodgkin’s lymphoma, and Burkitt lymphoma, while no lower-middle-income country achieved this survival goal for retinoblastoma, Wilms tumor, or LGG. In the upper-middle income category, 22% (n = 12/54) of all countries achieved at least 60% net survival for Wilms tumor, 46% (n = 25/54) for Burkitt lymphoma, 33% (n = 18/54) for Hodgkin’s lymphoma, 33% (n = 18/54) for ALL, and 28% (n = 15/54) for retinoblastoma. No upper-middle-income country achieved at least 60% net survival for LGG. All high-income countries achieved at least 60% net survival across all cancer tracers except for LGG, where only 5% (3/64) of high-income countries achieved this survival goal. Further analysis revealed that ‘less than 40% net survival’ was the most common pattern among countries not achieving the 2030 GICC goal at baseline, especially among low-income and lower-middle-income countries as well as the African, South-East Asia, and West Pacific regions (**S1 Fig**).

When stratified by WHO region, we found most of the countries in the European region achieved the goal of at least 60% net survival for Burkitt lymphoma (76%, n = 41/54), ALL (74%, n = 40/54), Hodgkin lymphoma (72%, n = /54), Wilms Tumor (70%, n = 38/54), and Retinoblastoma (69%, n = 37/54). In the American region, 61% (n = 22/36) of countries achieved this goal for Burkitt lymphoma, 50% (n = 18/36) for ALL and Hodgkin lymphoma, and 42% (n = 15/36) for Retinoblastoma and Wilms Tumor. The West Pacific region, South-East Asia region, Eastern Mediterranean region, and the African region reported the lowest number of countries attaining the survival goal across all cancer tracers, with the African region ranging from 2% (n = 1/50) to 4% (n = 2/50) of countries attaining this goal across all cancer traces, except for LGG.

**Fig 5:**
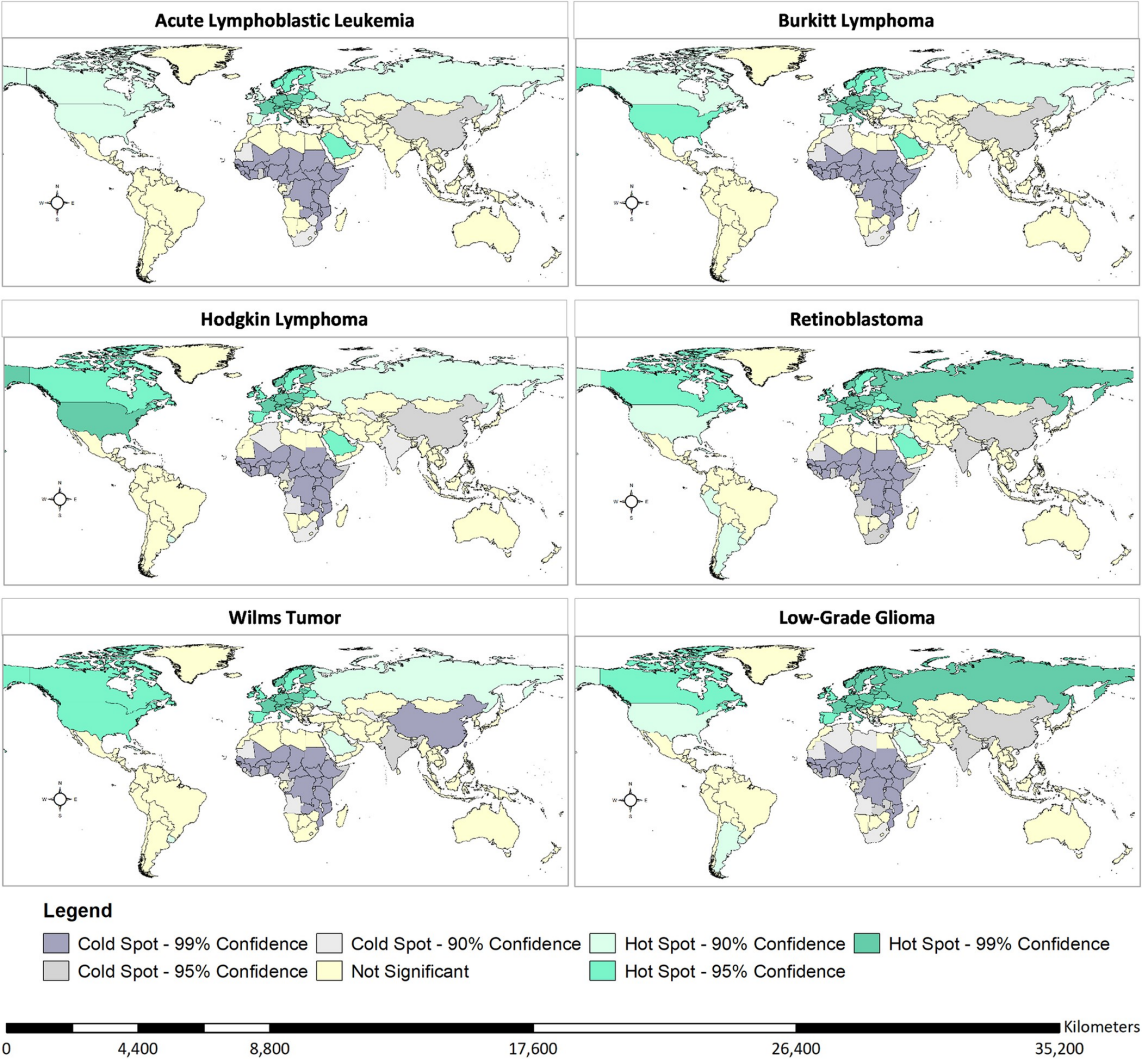
Hot spot analysis evaluating the geographic clusters with the highest and lowest childhood cancer 5-year (2015–2019) net survival per cancer tracer. Notes: Lymphoid was used as a proxy of ALL. Other gliomas were used as a proxy for Low-Grade Glioma. Hot spots (red) show clusters of data points with adjacent data points with high childhood survival. Cold spots (blue) show clusters with adjacent data points with low childhood survival. Base layer data available from the Humanitarian Data Exchange website: https://data.humdata.org/dataset/first-level-administrative-divisions-admin1.

Using hot spot analysis, we identified clusters of the highest net survival across all cancer tracer diagnoses concentrated in the global north, including North America and Europe (**Fig 5**). Clusters with the lowest net survival across all cancer tracers were concentrated in the global south, including Sub-Saharan Africa and South-East Asia. For retinoblastoma, three South American countries, Peru, Argentina, and Uruguay were identified among the countries with high survival. Uruguay was the only country in South America identified with the highest survival for Hodgkin’s lymphoma and Wilms tumor.

## Discussion

There are wide disparities in childhood cancer survival around the world, although the precise magnitude of disparities in survival between countries is difficult to ascertain. To address this gap, we used data from modeling estimates by Ward et al. to identify the 5-year net survival for childhood cancer overall and for each of the six GICC cancer tracer diagnoses for children, with the exception of ‘other gliomas’ used for LGG. We found wide disparities in survival for childhood cancer across countries and survival by cancer tracer diagnosis. Our study found that 92% of high-income countries and no low-income countries reached the 60% survival metric at baseline, and offers national survival baselines upon which progress can be monitored.

It is important to note that the GICC goal of 60% survival is not intended to be a discrete target for each individual country, but rather a global goal to be supported by all countries to the best of their capacity. Our analysis found that the lowest income settings are farthest away from the 60% goal, indicating the need for the most investment and scale-up capacity building to improve childhood cancer survival. However, countries closer to the 60% goal or slightly over that goal still can make improvements in their own survival for children with cancer. We strongly recommend that the 60% goal be viewed as a single global goal, with collective national responsibilities, similar to what has been described for the Sustainable Development Goals [17]. In light of the single global goal, since most of the investments aimed at improving childhood cancer survival will need to occur in countries with limited resources, we also recommend investments, solidarity, and collective responsibility towards the countries in most need. This will likely mean that high-income countries share, invest, and aid low-income countries equitably scale-up childhood cancer systems that they have already scaled themselves. The real aim is to improve childhood cancer survival for all children, regardless of where they live, to the optimum. Global collective responsibility will no doubt get us to our goals quicker and with a more equitable distribution of resources for all the world’s children.

Our analysis showed that low-income and lower-middle-income countries had the worst survival estimates for childhood cancer overall. These findings align with most existing literature which suggests that national economic status has a strong association with cancer outcomes for children [18, 19]. National wealth is closely associated with many health outcomes, with high-income countries having greater healthcare capacity and infrastructure to support high-quality cancer services, including advanced therapeutics and diagnostics [20]. Rapid diagnosis and access to care are critical to improve outcomes for childhood cancer in LMICs. Despite the challenges found in LMICs, gaining a deeper understanding of the factors contributing to variations in performance among peer countries is crucial for identifying practical insights. These lessons can encompass innovative strategies, adaptive practices, or unique approaches that contribute to success despite limited resources.

We identified Burkitt lymphoma as the leading tracer diagnosis in achieving the GICC target goal for survival. This might be in part because the treatment is highly cost-effective, with at least 50 percent of children with this disease and up to 70 percent of children with localized stage I or stage II disease being cured with generally accessible medications such as cyclophosphamide and methotrexate [21]. Furthermore, care can be delivered even in medical centers with lower levels of infrastructure compared to other cancer diagnoses [22].

In contrast, although retinoblastoma can be effectively treated when detected early, this cancer tracer had one of the worst performances, after the ‘other gliomas’ tracer, used as a proxy for LGG, with only 35% of countries achieving the GICC target goal. A study on a global cohort of children with retinoblastoma revealed a strong association between late presentation and the economic status of the country [23]. Subsequent longitudinal investigations exposed a significant survival disparity based on economic levels, with a nearly 17-fold difference between high-income and low-income countries [24]. Early detection plays a key role in increasing retinoblastoma survival and underscores the significance of early detection and timely treatment initiation. Moreover, underfunding in eye health is a persistent challenge in many countries [25].

The current net survival as reported in the modeling estimates by Ward et al. for LGG is exceedingly low and likely reflects challenges with data collection for this tumor. LGG was selected as the pediatric central nervous system (CNS) tumor by the GICC because of expected favorable outcomes and the role of multimodal therapy (chemotherapy, neurosurgery, and radiotherapy), and we refer readers to the detailed discussion by Moreira et al for further detail [26, 27]. As also discussed by Moreira et al., the International Classification of Childhood Cancer 3^rd^ edition does not clearly classify pediatric CNS tumors into clinically relevant groups. As currently conducted, LGG are aggregated in the categories of astrocytomas and other gliomas, inclusive of some high-grade lesions, and are currently impossible to analyze as a subgroup with existing classification schema. Given the widely variable clinical activity and survival of this broad group of CNS tumors, it is impossible to track the survival of LGG using this classification system alone, and our analysis confirmed these challenges with current tracking metrics and likely underestimated the survival of LGG alone.

Although a country’s income level is a marker of a strong health system for cancer, many of these services specific to children require specialized care, such as pediatric surgeons for biopsies, chemotherapy, well-defined and timely referral to pediatric tertiary treatment centers, and child-centered supportive care to minimize risks of abandonment. As well, identification of system-level factors that impact cancer outcomes for children can help develop strategic policies and interventions [28]. Individually, each point along the cancer care continuum, such as diagnosis, treatment, and continuation of treatment impacts outcomes. However, the interplay of these timepoints likely impact the outcome more as they can compound on one another if delays occur.

### Limitations

Our study has several additional limitations beyond those already discussed above. First, the estimates for childhood cancer net survival were obtained from a simulation-based model reported by Ward et al., [14] which accounted for several clinical, epidemiologic, health system, and country-level factors such as income level group. This limitation has been addressed in the original modeling study by comparing the simulated data to existing observable data from cancer population registries, with a 95% overlap of confidence interval results for 99% of all predictions [14]. Furthermore, even when hierarchical models allow for ’partial pooling’/’borrowing’ there is still space for country-level variation within groups, especially where empirical estimates exist for specific countries. Second, because of data availability limitations, we used lymphoid and ‘other gliomas’ data as proxy diagnoses for ALL and LGG. The outcomes for all lymphoid malignancies may underestimate the survival for ALL itself. As discussed above, the use of ‘other gliomas’ likely underestimates the true survival for LGG. Third, the GICC target is for children 0–19 years of age, but the Lancet Oncology Commission analyses and the survival estimates used in this study were for children 0–14 and thus did not include all adolescent cancers. Fourth, we used the 2019 World Bank income level classifications rather than the 2015 classifications. Although our survival estimates were based on the years 2015 to 2019, only 18 countries changed income classification levels during that time (Armenia, Benin, Comoros, Indonesia, Kosovo, Mauritius, Palau, Panama, Romania, Samoa, Senegal, Sudan, Syrian Arab Republic, Tajikistan, Tanzania, Tonga, Yemen, and Zimbabwe). Finally, some of our data comes from nations with unique political contexts and limited data collection infrastructure. For example, it is well known that Yemen and the Syrian Arab Republic have a long history of civil conflicts and other significant challenges for data infrastructure, which were not accounted for in the Ward modeling approach [29].

Perhaps the most notable limitation in our study is the use of modeled estimates for 5-year childhood cancer net survival from 2015 to 2019, while the GICC was launched in 2018. We recognize that many countries have made significant strides in improving childhood cancer outcomes since 2018 due to the implementation of GICC strategies. Our analysis is intended to serve as a baseline for reaching the GICC goals, rather than an indication of a country’s improvement since the onset of GICC. Furthermore, our results should not replace the importance of countries building capacity to directly measure survival which is a better measure of baseline survival than modeled estimates.

### Policy recommendations

In order to directly measure survival for children with cancer, we advocate for directly measuring cancer survival for children through existing metrics such as the ongoing CONCORD and SURVCAN studies [30, 31], expanding the number and types of data collected by population-based registries in LMICs with regards to childhood cancers, and utilizing innovative solutions supported by the CUREALL framework, such as hospital-based registries as a proxy for surveillance tool in the absence of population-based registries and to address the inherent several-year lag in population-based cancer registry data collection. Moving forward, real-time, observational data is needed to clearly observe gaps in children’s survival from cancer, particularly in areas with the lowest survival. Monitoring of prospective data in addition to interventions, such as those outlined in the CUREALL framework, will allow us to identify causal relationships impacting survival. In other words, directly observed survival data rather than modeled estimates will allow us to see in real time what interventions are working or not. In doing so, our future efforts will continue to be more refined, targeted, and effective.

## Conclusions

A majority of LMICs have not yet attained the GICC target goal of at least 60% survival for childhood cancer in 2019, with variable success for the six childhood cancer tracer diagnoses of the GICC. As countries around the world continue to work to improve survival from childhood cancer to attain these goals, our analyses provide further epidemiologic context to support implementation of the 4 pillars and 3 enablers laid out in the GICC CUREALL framework as well as six actions called for by the Lancet Oncology Commission for Sustainable Care for Children with Cancer.

## Supporting information

S1 Table Status of childhood cancer 5-year (2015–2019) net survival overall and 5-year (2015–2019) net survival for each of the six GICC cancer tracer diagnoses at baseline by country. (DOCX)

S1 Fig Status of childhood cancer 5-year (2015–2019) net survival at baseline for each of the six cancer tracer diagnoses and stratified made by WHO region and World Bank income level. Note: Survival was categorized into at least 80%, from 60% to 79%, from 40% to 59%, and less than 40%. ALL = Acute Lymphoblastic Leukemia. Lymphoid was used as a proxy of ALL. Other gliomas were used as a proxy for Low-Grade Glioma.
(TIF)
