# Supplementary material for: Towards attainment of the 2030 goal for childhood cancer survival for the World Health Organization Global Initiative for Childhood Cancer: An ecological, cross-sectional study
Source: PLOS Glob Public Health. 2024 Aug 19;4(8):e0002530. doi: 10.1371/journal.pgph.0002530 (PMC11332931; doi:10.1371/journal.pgph.0002530)
Supplement: S1 Table — (DOCX) [file pgph.0002530.s001.docx]

**S1 Table.** **Status of childhood cancer 5-year (2015-2019) net survival overall and 5-year (2015-2019) net survival for each of the six GICC cancer tracer diagnoses at baseline by country.**

| **Country** | **2030 GICC initial goal: at least 60% survival** | | | | | | |
| --- | --- | --- | --- | --- | --- | --- | --- |
|  | **Survival overall** | **ALL** | **Hodgkin** | **Burkitt** | **Retinoblastoma** | **Wilms Tumor** | **LLG** |
| Afghanistan | no | no | no | no | no | no | no |
| Angola | no | no | no | no | no | no | no |
| Albania | no | yes | no | yes | no | no | no |
| Andorra | yes | yes | yes | yes | yes | yes | no |
| United Arab Emirates | yes | yes | yes | yes | yes | yes | no |
| Argentina | yes | yes | yes | yes | yes | yes | no |
| Armenia | no | no | no | no | no | no | no |
| Antigua and Barbuda | yes | yes | yes | yes | yes | yes | no |
| Australia | yes | yes | yes | yes | yes | yes | no |
| Austria | yes | yes | yes | yes | yes | yes | no |
| Azerbaijan | no | no | no | no | no | no | no |
| Burundi | no | no | no | no | no | no | no |
| Belgium | yes | yes | yes | yes | yes | yes | yes |
| Benin | no | no | no | no | no | no | no |
| Burkina Faso | no | no | no | no | no | no | no |
| Bangladesh | no | no | no | no | no | no | no |
| Bulgaria | yes | yes | yes | yes | yes | yes | no |
| Bahrain | yes | yes | yes | yes | yes | yes | no |
| Bahamas | yes | yes | yes | yes | no | yes | no |
| Bosnia and Herzegovina | no | no | no | no | no | no | no |
| Belarus | yes | yes | yes | yes | yes | yes | no |
| Belize | no | no | no | no | no | no | no |
| Bermuda | yes | yes | yes | yes | yes | yes | no |
| Bolivia (Plurinational State of) | no | no | no | no | no | no | no |
| Brazil | no | yes | yes | yes | yes | yes | no |
| Barbados | yes | yes | yes | yes | yes | yes | no |
| Brunei Darussalam | yes | yes | yes | yes | yes | yes | no |
| Bhutan | no | no | no | no | no | no | no |
| Botswana | no | no | no | no | no | no | no |
| Central African Republic | no | no | no | no | no | no | no |
| Canada | yes | yes | yes | yes | yes | yes | no |
| Switzerland | yes | yes | yes | yes | yes | yes | yes |
| Chile | yes | yes | yes | yes | yes | yes | no |
| China | no | no | yes | yes | no | no | no |
| Côte d'Ivoire | no | no | no | no | no | no | no |
| Cameroon | no | no | no | no | no | no | no |
| Democratic Republic of the Congo | no | no | no | no | no | no | no |
| Congo | no | no | no | no | no | no | no |
| Colombia | yes | yes | yes | yes | yes | yes | no |
| Comoros | no | no | no | no | no | no | no |
| Cabo Verde | no | no | no | no | no | no | no |
| Costa Rica | yes | yes | yes | yes | yes | yes | no |
| Cuba | yes | yes | yes | yes | yes | yes | no |
| Cyprus | yes | yes | yes | yes | yes | yes | no |
| Czechia | yes | yes | yes | yes | yes | yes | no |
| Germany | yes | yes | yes | yes | yes | yes | no |
| Djibouti | no | no | no | no | no | no | no |
| Dominica | no | no | no | yes | no | no | no |
| Denmark | yes | yes | yes | yes | yes | yes | no |
| Dominican Republic | no | no | no | no | no | no | no |
| Algeria | no | yes | yes | yes | no | no | no |
| Ecuador | no | no | yes | yes | yes | no | no |
| Egypt | no | no | no | no | no | no | no |
| Eritrea | no | no | no | no | no | no | no |
| Spain | yes | yes | yes | yes | yes | yes | no |
| Estonia | yes | yes | yes | yes | yes | yes | no |
| Ethiopia | no | no | no | no | no | no | no |
| Finland | yes | yes | yes | yes | yes | yes | no |
| Fiji | no | no | no | no | no | no | no |
| France | yes | yes | yes | yes | yes | yes | no |
| Micronesia (Federated States of) | no | no | no | no | no | no | no |
| Gabon | no | no | no | no | no | no | no |
| United Kingdom | yes | yes | yes | yes | yes | yes | no |
| Georgia | no | no | no | no | no | no | no |
| Ghana | no | no | no | no | no | no | no |
| Guinea | no | no | no | no | no | no | no |
| Gambia | no | no | no | no | no | no | no |
| Guinea-Bissau | no | no | no | no | no | no | no |
| Equatorial Guinea | no | no | no | no | no | no | no |
| Greece | yes | yes | yes | yes | yes | yes | no |
| Grenada | no | no | no | no | yes | no | no |
| Greenland | yes | yes | yes | yes | yes | yes | no |
| Guatemala | no | no | no | no | no | no | no |
| Guyana | no | no | no | yes | no | no | no |
| Honduras | no | no | no | no | no | no | no |
| Croatia | yes | yes | yes | yes | yes | yes | no |
| Haiti | no | no | no | no | no | no | no |
| Hungary | yes | yes | yes | yes | yes | yes | no |
| Indonesia | no | no | no | no | no | no | no |
| India | no | no | no | no | no | no | no |
| Ireland | yes | yes | yes | yes | yes | yes | no |
| Iran (Islamic Republic of) | no | yes | no | yes | no | no | no |
| Iraq | no | no | no | no | no | no | no |
| Iceland | yes | yes | yes | yes | yes | yes | no |
| Israel | yes | yes | yes | yes | yes | yes | no |
| Italy | yes | yes | yes | yes | yes | yes | no |
| Jamaica | no | no | no | no | no | no | no |
| Jordan | yes | yes | yes | yes | yes | yes | no |
| Japan | yes | yes | yes | yes | yes | yes | no |
| Kazakhstan | no | no | no | no | no | no | no |
| Kenya | no | no | no | no | no | no | no |
| Kyrgyzstan | no | no | no | no | no | no | no |
| Cambodia | no | no | no | no | no | no | no |
| Kiribati | no | no | no | no | no | no | no |
| Saint Kitts and Nevis | yes | yes | yes | yes | no | yes | no |
| Republic of Korea | yes | yes | yes | yes | yes | yes | no |
| Kuwait | yes | yes | yes | yes | yes | yes | no |
| Lao People's Democratic Republic | no | no | no | no | no | no | no |
| Lebanon | no | yes | yes | yes | no | no | no |
| Liberia | no | no | no | no | no | no | no |
| Libya | no | no | no | no | no | no | no |
| Saint Lucia | no | no | no | no | no | no | no |
| Sri Lanka | no | no | no | no | no | no | no |
| Lesotho | no | no | no | no | no | no | no |
| Lithuania | yes | yes | yes | yes | yes | yes | no |
| Luxembourg | yes | yes | yes | yes | yes | yes | no |
| Latvia | yes | yes | yes | yes | yes | yes | no |
| Morocco | no | no | no | no | no | no | no |
| Monaco | yes | yes | yes | yes | yes | yes | no |
| Republic of Moldova | no | no | no | no | no | no | no |
| Madagascar | no | no | no | no | no | no | no |
| Maldives | no | no | no | no | no | no | no |
| Mexico | no | no | yes | yes | no | no | no |
| Marshall Islands | no | no | no | no | no | no | no |
| North Macedonia | no | no | no | no | no | no | no |
| Mali | no | no | no | no | no | no | no |
| Malta | no | yes | yes | yes | yes | yes | no |
| Myanmar | no | no | no | no | no | no | no |
| Montenegro | no | yes | yes | yes | no | no | no |
| Mongolia | no | no | no | no | no | no | no |
| Mozambique | no | no | no | no | no | no | no |
| Mauritania | no | no | no | no | no | no | no |
| Mauritius | no | no | no | no | no | no | no |
| Malawi | no | no | no | no | no | no | no |
| Malaysia | yes | yes | yes | yes | yes | yes | no |
| Namibia | no | no | no | no | no | no | no |
| Niger | no | no | no | no | no | no | no |
| Nigeria | no | no | no | no | no | no | no |
| Nicaragua | no | no | no | no | no | no | no |
| Netherlands | yes | yes | yes | yes | yes | yes | no |
| Norway | yes | yes | yes | yes | yes | yes | no |
| Nepal | no | no | no | no | no | no | no |
| Nauru | yes | yes | yes | yes | no | yes | no |
| New Zealand | yes | yes | yes | yes | yes | yes | no |
| Oman | yes | yes | yes | yes | no | yes | no |
| Pakistan | no | no | no | no | no | no | no |
| Panama | no | yes | no | yes | no | no | no |
| Peru | no | yes | yes | yes | yes | no | no |
| Philippines | no | no | no | no | no | no | no |
| Palau | no | no | no | no | no | no | no |
| Papua New Guinea | no | no | no | no | no | no | no |
| Poland | yes | yes | yes | yes | yes | yes | no |
| Puerto Rico | yes | yes | yes | yes | yes | yes | no |
| Democratic People's Republic of Korea | no | no | no | no | no | no | no |
| Portugal | yes | yes | yes | yes | yes | yes | no |
| Paraguay | no | yes | no | yes | no | no | no |
| Palestine | no | no | no | no | no | no | no |
| Qatar | yes | yes | yes | yes | yes | yes | no |
| Romania | no | no | no | no | no | no | no |
| Russian Federation | yes | yes | yes | yes | yes | yes | no |
| Rwanda | no | no | no | no | no | no | no |
| Saudi Arabia | yes | yes | yes | yes | no | yes | no |
| Sudan | no | no | no | no | no | no | no |
| Senegal | no | no | no | no | no | no | no |
| Singapore | yes | yes | yes | yes | yes | yes | no |
| Solomon Islands | no | no | no | no | no | no | no |
| Sierra Leone | no | no | no | no | no | no | no |
| El Salvador | no | no | no | no | no | no | no |
| San Marino | yes | yes | yes | yes | no | yes | no |
| Somalia | no | no | no | no | no | no | no |
| Serbia | no | no | no | yes | no | no | no |
| South Sudan | no | no | no | no | no | no | no |
| Sao Tome and Principe | no | no | no | no | no | no | no |
| Suriname | no | no | no | no | no | no | no |
| Slovakia | yes | yes | yes | yes | yes | yes | no |
| Slovenia | yes | yes | yes | yes | yes | yes | no |
| Sweden | yes | yes | yes | yes | yes | yes | yes |
| Eswatini | no | no | no | no | no | no | no |
| Seychelles | yes | yes | yes | yes | yes | yes | no |
| Syrian Arab Republic | no | no | no | no | no | no | no |
| Chad | no | no | no | no | no | no | no |
| Togo | no | no | no | no | no | no | no |
| Thailand | no | yes | yes | yes | yes | yes | no |
| Tajikistan | no | no | no | no | no | no | no |
| Turkmenistan | no | no | no | no | no | no | no |
| Timor-Leste | no | no | no | no | no | no | no |
| Tonga | no | no | no | no | no | no | no |
| Trinidad and Tobago | yes | yes | yes | yes | no | yes | no |
| Tunisia | no | no | no | no | no | no | no |
| Turkey | yes | yes | yes | yes | yes | yes | no |
| Tuvalu | no | no | no | yes | no | no | no |
| Taiwan (Province of China) | yes | yes | yes | yes | yes | yes | no |
| United Republic of Tanzania | no | no | no | no | no | no | no |
| Uganda | no | no | no | no | no | no | no |
| Ukraine | no | no | no | no | no | no | no |
| Uruguay | yes | yes | yes | yes | yes | yes | no |
| United States of America | yes | yes | yes | yes | yes | yes | no |
| Uzbekistan | no | no | no | no | no | no | no |
| Saint Vincent and the Grenadines | no | no | no | no | no | no | no |
| Venezuela (Bolivarian Republic of) | no | no | no | no | no | no | no |
| Viet Nam | no | no | no | no | no | no | no |
| Vanuatu | no | no | no | no | no | no | no |
| Samoa | no | no | no | no | no | no | no |
| Yemen | no | no | no | no | no | no | no |
| South Africa | no | no | no | no | no | no | no |
| Zambia | no | no | no | no | no | no | no |
| Zimbabwe | no | no | no | no | no | no | no |
