# Supplementary figures and images for: Towards attainment of the 2030 goal for childhood cancer survival for the World Health Organization Global Initiative for Childhood Cancer: An ecological, cross-sectional study
Source: PLOS Glob Public Health. 2024 Aug 19;4(8):e0002530. doi: 10.1371/journal.pgph.0002530 (PMC11332931; doi:10.1371/journal.pgph.0002530)

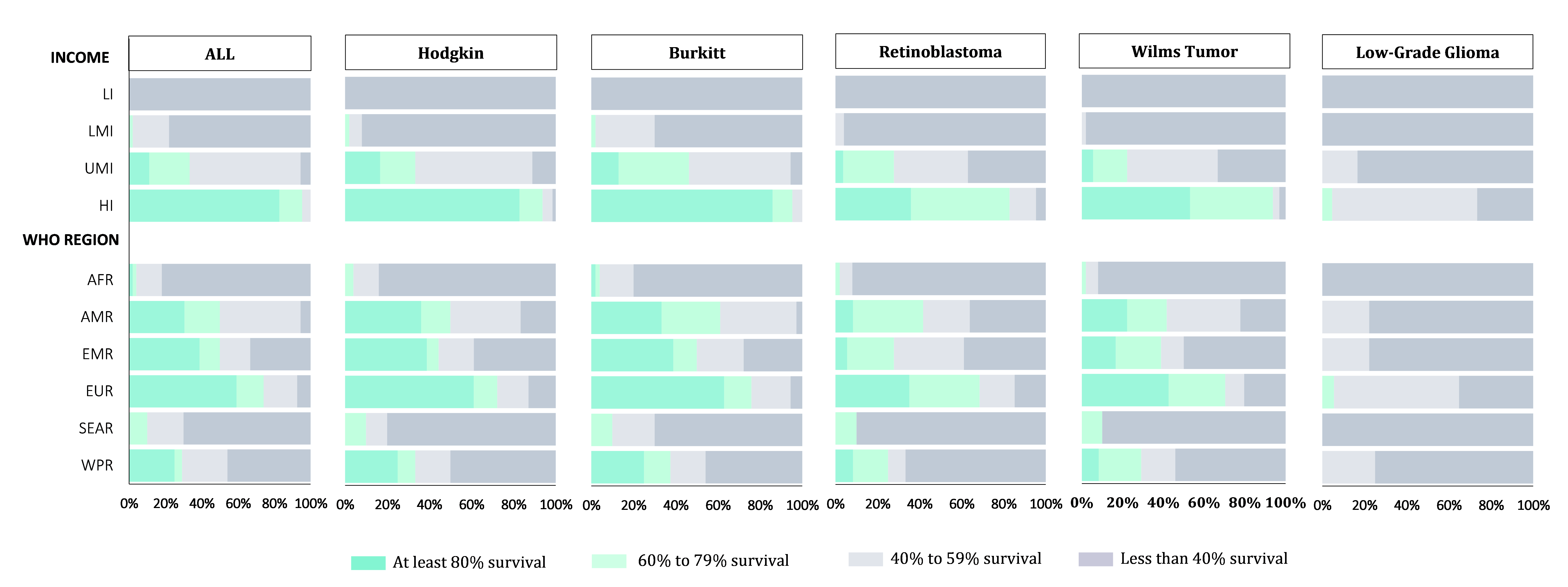

Supplement: S1 Fig — Note: Survival was categorized into at least 80%, from 60% to 79%, from 40% to 59%, and less than 40%. ALL = Acute Lymphoblastic Leukemia. Lymphoid was used as a proxy of ALL. Other gliomas were used as a proxy for Low-Grade Glioma. (TIF) [file pgph.0002530.s002.tif]
